# Motor Nerve Conduction Block Estimation in Demyelinating Neuropathies by Deconvolution

**DOI:** 10.3390/bioengineering9010023

**Published:** 2022-01-10

**Authors:** Luca Mesin, Edoardo Lingua, Dario Cocito

**Affiliations:** 1 Mathematical Biology and Physiology, Department Electronics and Telecommunications, Politecnico di Torino, 10129 Turin, Italy; s261655@studenti.polito.it; 2 S.C. Neurologia I, Dipartimento di Neuroscienze, Universitá di Torino, 10124 Torino, Italy; dariococito@yahoo.it; 3 I.R.C.C.S. Istituti Clinici Scientifici, Fondazione S. Maugeri, 27100 Pavia, Italy

**Keywords:** CMAP, CIDP, MMN, motor response, nerve conduction study, temporal dispersion, conduction block

## Abstract

A deconvolution method is proposed for conduction block (CB) estimation based on two compound muscle action potentials (CMAPs) elicited by stimulating a nerve proximal and distal to the region in which the block is suspected. It estimates the time delay distributions by CMAPs deconvolution, from which CB is computed. The slow afterwave (SAW) is included to describe the motor unit potential, as it gives an important contribution in case of the large temporal dispersion (TD) often found in patients. The method is tested on experimental signals obtained from both healthy subjects and pathological patients, with either Chronic Inflammatory Demyelinating Polyneuropathy (CIDP) or Multifocal Motor Neuropathy (MMN). The new technique outperforms the clinical methods (based on amplitude and area of CMAPs) and a previous state-of-the-art deconvolution approach. It compensates phase cancellations, allowing to discriminate among CB and TD: estimated by the methods of amplitude, area and deconvolution, CB showed a correlation with TD equal to 39.3%, 29.5% and 8.2%, respectively. Moreover, a significant decrease of percentage reconstruction errors of the CMAPs with respect to the previous deconvolution approach is obtained (from a mean/median of 19.1%/16.7% to 11.7%/11.2%). Therefore, the new method is able to discriminate between CB and TD (overcoming the important limitation of clinical approaches) and can approximate patients’ CMAPs better than the previous deconvolution algorithm. Then, it appears to be promising for the diagnosis of demyelinating polyneuropathies, to be further tested in the future in a prospective clinical trial.

## 1. Introduction

Electrodiagnostic (EDX) examination provides important information on the peripheral nervous system (PNS) functionality that completes and extends the findings of routine neurologic tests [1]. It is built upon two clinical techniques, the nerve conduction studies (NCSs; focused on either sensory or motor nerves) and the needle electrode examination (NEE), which are usually combined to provide complementary information of the PNS [2]. EDX examination is usually focused on the localization of a lesion and its characterization, in terms of severity, rate of progression and pathophysiological features.

In this work, we are interested in motor NCSs focused on demyelinating conduction block (CB), i.e., the failure of an action potential (AP) to propagate through an intact axon of a motoneuron [3,4]. It is important to distinguish it from axonal loss, in which the axon is disrupted, so that its distal portion is no longer connected to the cell body and undergoes a process called Wallerian degeneration [5]. On the other hand, the lesions of the myelin sheaths (possibly caused by auto-antibodies against antigens at the node of Ranvier [6]) determine a slowing of AP propagation along the nerve (with abnormal temporal dispersion (TD) [7]) and sometimes the block of impulses (i.e., a CB [8]), reflected into muscle weakness. The methods routinely used in clinical practice for CB estimation are based on the comparison of the amplitudes or the areas of two compound muscle action potentials (CMAPs) elicited with transcutaneous electrical nerve stimulation at sites proximal and distal to the nerve segment in which the CB is suspected [4,9]. CB estimation obtained with these standard clinical methods is altered by the phase cancellations produced by TD of the motor unit (MU) APs (MUAPs) constituting the CMAPs. Indeed, both CB and TD may result in variations of amplitude and area among distal and proximal CMAPs [1]. These variations depend on the length of the nerve segment, as proved in experiments [10] and simulations [11,12,13,14]. Abnormal TD influences amplitude and area due to important phase cancellations that mainly affect the proximal CMAP [12]. Thus, a large confidence threshold in the decrease of amplitude and area of distal versus proximal CMAPs (50% [12]) has been proposed to diagnose a CB. In order to face this problem, different works have suggested to combine information on both TD and amplitude or area decrease [15,16]. Different clinical criteria for CB estimation have been proposed [17]: for example, a drop in distal versus proximal CMAP peak-to-peak amplitude or area larger than 20% with less than a 15% variation of the duration [9] or different ranges of reductions of amplitude and area (from >30% to >60%) depending on the specific nerve and on TD (considering different cases if it is larger or smaller than 30%; Table 1 in [18]).

As an alternative, a method to compensate for TD was proposed in [19]. It was validated on simulations and experimental data from healthy subjects. It was able to compensate for different TDs, either simulated or induced in experiments by studying three different stimulation sites (and thus different conduction lengths). The method provides by deconvolution the delay distributions which, convolved with a representative waveform named kernel, optimally reconstruct the distal and the proximal CMAPs. This allows to ideally compensate for phase cancellations, discriminating CB and TD better than the approaches based on amplitude or area.

However, the method proposed in [19] did not include the slow afterwave (SAW) of muscle potentials [20,21,22,23,24,25]. In the case of small TD (as in healthy subjects), SAW contributes at the end of the CMAPs and could be removed by windowing. On the other hand, the TD is abnormal in subjects with demyelinating diseases, so that SAW could contribute to important phase cancellations in the proximal CMAP. This effectively rendered the previous method unusable on patients, i.e., exactly where it would have been needed. Thus, with the intent of extending the promising results shown in [19] to neuropathic patients with abnormal TD of the APs, in this paper SAW has been included in the deconvolution kernel. The method was then tested on experimental signals from both healthy subjects and pathological patients, with either Chronic Inflammatory Demyelinating Polyneuropathy (CIDP [1,7,26,27,28,29,30]) or Multifocal Motor Neuropathy (MMN [26,28,29,31,32]). Our results are expected to contribute to the discussion on guidelines [7,27,30] and the proposal of innovative methods for the assessment of CB [1,31].

## 2. Methods

### 2.1. Mathematical Model and Processing

The mathematical model and the CB estimation method follow the approach described in [19]. Specifically, the two CMAPs are represented as the sum of MUAPs, assumed to have the same shape, but different amplitudes and time lags. The prototype shape of the MUAPs is called convolution kernel. Thus, each CMAP is approximated as

(1) $$x\left(t\right)≃\sum_{n=1}^{N}A_{n}K\left(t-\tau_{n}\right)$$

where x(t) is the CMAP, ≃ stands for “approximately equal”, *N* is the number of contributions, K(t) is the kernel, $A_{n}$ is the amplitude of the *n*th MUAP, and $\tau_{n}$ its delay. Notice that the model can only approximate (and not fit exactly) a CMAP, as MUAPs cannot be represented accurately by a single kernel as they have different waveforms (depending on many factors, including muscle fibre anatomy, location and spreads of innervation zone and tendons, position of the MU, conduction velocity (CV), etc. [33]). Moreover, the recorded CMAPs include noise superimposed to the physiological bioelectric signal.

Equation (1) can be written as the following convolution equation:

(2) $$x\left(t\right)=K\left(t\right)*\sum_{n=1}^{N}A_{n}\delta \left(t-\tau_{n}\right)=K\left(t\right)*D\left(t\right)$$

where ∗ indicates convolution and the delay distribution D(t) is written as the sum of Dirac delta functions δ(t) delayed in time and scaled in amplitude $D\left(t\right)=\sum_{n=1}^{N}A_{n}\delta \left(t-\tau_{n}\right)$.

Notice that phase cancellations affect only the signal x(t), not the delay distribution, which is a non-negative function, indicating, for each time lag, the amplitude of the contributions superimposed to form the CMAP. Thus, a method able to estimate the delay distributions of MUAPs constituting the distal and proximal CMAPs and using this information to compute the CB is ideally insensitive to phase cancellations. The CB estimator proposed in [19] is indeed dependent only on the delay distributions $x^{prox}$ and $x^{dist}$ of the proximal and distal CMAPs:

(3) $$CB=1-\frac{\int x^{prox}\left(t\right)dt}{\int x^{dist}\left(t\right)dt}$$

where the integral symbol indicates the area under the curve (i.e., for the problem at hand, it is ideally proportional to the sum of the amplitudes of MUAPs contributing to the CMAPs). Thus, if the same contributions constitute the two CMAPs (only with different time lags), the two integrals in (3) have the same value and the estimated CB is zero. On the other hand, if the integral of the proximal delay distribution is lower than that of the distal one, it means that some contributions (i.e., some MUAPs) are absent in the proximal CMAP, due to a block of the corresponding APs. Notice that the proposed estimation of CB is affected by MUAP amplitude (so that the loss of a MUAP with larger amplitude gives a larger value of estimated CB than the loss of a small MUAP), which depends on MU size and location. However, this problem afflicts also amplitude and area approaches, which are also affected by phase cancellations. On the other hand, the proposed estimation of CB has the merit of being ideally insensitive to phase cancellations and provided important improvements over standard approaches based on CMAP amplitude or area, both in simulations and experiments on healthy subjects [19].

In order to apply the CB estimation in Equation (3), the delay distributions for the proximal and distal CMAPs should be computed. This requires to estimate the kernel and invert the convolution operator in Equation (2), thus making a deconvolution. This is an inverse problem [34,35,36,37], requiring regularization approaches to stabilize the solution and the imposition of a-priori information (e.g., small energy of the solution to avoid excessive phase cancellations, limited time range of the possible lags reflecting physiological values of CV, non-negativity of the delay distribution [19]).

The kernel was estimated as in [19], requiring that the same waveform could be used to reconstruct the two CMAPs with minimum sum of the mean squared errors (MSE). However, in [19] the kernel was given as the sum of few associated Hermite (AH) functions, as they are sufficient to approximately fit a biphasic (single differential) EMG. Here, an exponential decay was also included to account for the SAW, still keeping the same optimization approach. This additional term is described below.

An initial estimation of the exponential decay accounting for the SAW was obtained considering the distal CMAP (i.e., the one for which phase cancellations are less important, so that a biphasic shape followed by the SAW could be assumed). The SAW was assumed to be located after the two phases, so that the portion of CMAP following its minimum value was considered. Within such a time range (i.e., from the sample corresponding to the minimum of the CMAP to its end), the point (indicated as $p_{SAW}$) with most abrupt change was considered as the beginning of the SAW (specifically, $p_{SAW}$ spits the signal into two regions, for which the sum of the residual squared errors from their local means is minimal). An exponential decaying function was then fit to the CMAP queue, starting from $p_{SAW}$ (using a nonlinear programming method based on simplex search to select the amplitude and decay rate, i.e., parameters *A* and τ in the Equation (4) shown below). In order to remove the contribution of this exponential function before $p_{SAW}$, it was multiplied by a sigmoid function (which should be close to 0 before $p_{SAW}$ and to 1 after it, with a small transition region). The sigmoid was chosen in order to optimally fit the CMAP with AH functions after SAW removal (selecting the location and extension of the transition region, i.e., parameters *c* and *a* of Equation (5) shown below, by a sequential quadratic programming approach, constraining *c* to be within the range of the signal and *a* to be positive and lower than 1).

Then, the initial kernel was the sum of the projection of the distal CMAP on the first six AH functions and of the exponential decay detailed above (notice that only the first contribution was included in [19], neglecting the exponential decay). The parameters were then updated as in [19] by searching iteratively the minimum in the direction of the gradient of the MSE in reconstructing the two CMAPs by convolutions with the same kernel (constraining the delay distributions to be positive and within supports imposed by physiological limits in nerve CV). Eleven parameters were identified:

- seven parameters related to the AH functions (the scaling factor and the amplitude of the six functions; these are the only parameters considered in [19]);
- two parameters defining the descending exponential e(t), namely, the amplitude *A* and the time constant τ
  (4) $$e\left(t\right)=Ae^{-\frac{t}{\tau }}$$
- two parameters defining the sigmoid function s(t), i.e., the time instant *c* in which s(t)=0.5 and the rate of change *a*
  (5) $$s\left(t\right)=\frac{1}{1+e^{-a(t-c)}}$$

### 2.2. Experimental Data

Both healthy controls and pathological patients were included in the study, following the principles of the Declaration of Helsinki. The healthy subjects were the same as those in [19] (age, mean ± SD, 28 ± 5 years; stature, 179 ± 7 cm; weight, 72 ± 4 kg), but some additional traces were considered. The stimulation current was supra-maximal, biphasic, square, 0.1 ms long and obtained using a Viking Select device (Nicolet Biomedical Inc. Madison, WI, USA). After ulnar nerve stimulation, surface EMG signals were acquired from the right abductor digiti minimi muscle (belly-tendon recording [38]). The proximal site of stimulation was above-elbow while the distal site was either below-elbow or at the wrist (experimental protocol as in [39]). The distances (mean ± SD) of wrist, below-elbow and above-elbow from the motor point were 78.5 ± 3.7 mm, 325.4 ± 18.4 mm and 424.2 ± 17.4 mm, respectively. Motor responses were filtered between 2 Hz and 10 kHz; skin temperature was maintained above 35 °C.

A retrospective study was performed on CIDP and MMN patients (followed from a clinical, electrophysiological and therapeutic point of view by D.C., following the normal clinical practice carried out at “I.R.C.C.S. Istituti Clinici Scientifici, S. Maugeri Foundation, Pavia, Italy”), which are part of a more relevant case series contained in the Italian CIDP Database and Italian MMN Database [7,27]. Diagnostic criteria were defined in [40] for CIDP and in [41] for MMN. As part of the diagnostic work up of each patient, the presence of a Martin–Gruber anastomosis was excluded (indeed, in the presence of this kind of anastomosis, the CMAP amplitude of the ulnar nerve at elbow might appear significantly reduced if compared to the one recorded from distal stimulation, leading to interpretation errors in the nerve conduction study [42].

Most of the data were kept in medical records, printed on paper. They were manually digitized by a custom routine in Matlab from scanned versions of the clinical records. Different nerve segments have been measured: median nerve, with stimulations at wrist and elbow; ulnar nerve with stimulation sites at either wrist and above elbow or below and above elbow; and peroneal nerve with stimulation at either ankle and poplite or fibula and poplite. Information on conduction distances and nerve CV were not always available; then, when not available, the following distances were assumed as preliminary reference and then either adjusted (if CV was available) or fine-tuned in order that the delay distributions were reasonable: 7–10 cm from the distal stimulation site to the first electrode of the bipolar recording system; conduction distance was assumed 25 cm for the median nerve; 35 cm and 10 cm were used for ulnar nerve when considering either wrist and above elbow or below and above elbow, respectively; 44 cm and 10 cm were considered for peroneal nerve with stimulation at either ankle and poplite or fibula and poplite, respectively.

In total, our dataset included 26 pairs of CMAPs from healthy controls (considering also repeated measures on the same 10 subjects, which were neglected in [19]), 65 from 23 CIDP patients and 26 from 8 MMN patients.

### 2.3. Statistical Analysis

Data (i.e., TD and CB) were represented in box and whiskers plots, showing median, quartiles and range (with outliers indicated individually). Moreover, the correlation between CB (estimated by different methods, i.e., amplitude, area and deconvolution) and TD was computed using the Pearson’s coefficient. The performances of the estimations of the CMAPs using our model Equation (2) was measured in terms of the root mean squared error. In order to give an indication of discrimination among groups when CB was estimated using different approaches, we used the Fisher’s ratio, i.e., the square of the difference of the means divided by the sum of variances.

Non parametric Scheirer–Ray–Hare two-way analysis of variance (ANOVA) [43] was performed to check possible effects in CB estimation of the different approaches (amplitude, area and deconvolution) and pathology (control, CIDP and MMN). Wilcoxon signed rank test for paired comparisons was applied as post hoc for different estimation methods. To compare the estimations from patients with different pathologies, the Wilcoxon rank sum test was applied.

## 3. Results

Amplitude, area and deconvolution approaches have been applied to the CMAPs of our dataset and their estimations of CB have been compared.

**Figure 1:**
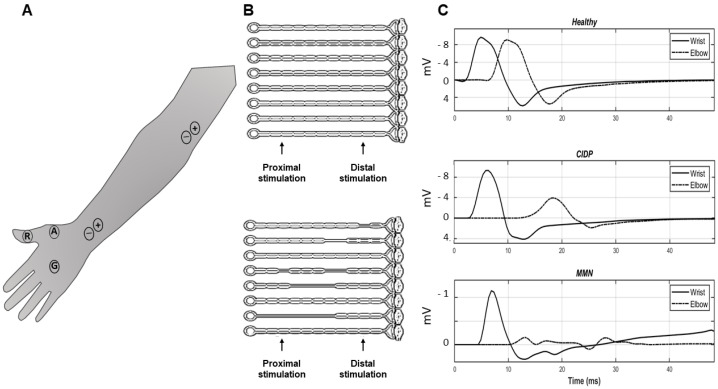
Motor nerve conduction study of the Median Thenar (MT) muscle: comparison between controls and patients with segmental demyelination. (**A**) Motor responses are recorded by surface electrodes (A and R) and the ground electrode G is used for reference. The nerve is depolarized via external stimulation at two distinct sites (proximal and distal pairs of electrodes for bipolar stimulation are indicated). (**B**) Model of conduction block: intact axons on top and multifocal demyelination on bottom, typical of acquired demyelinating neuropathies (e.g., CIDP and MMN). (**C**) Experimental signals recorded from the MT of different subjects: healthy (top row), CIDP (middle row) and MMN (bottom row).

Figure 1 shows a representation of a motor nerve conduction study and some examples of signals. In A, proximal and distal stimulation of the median nerve, innervating the thenar muscles, is considered. Surface electrodes (A and R) are placed over the muscle belly and tendon (respectively) to record the CMAPs in single differential configuration. Moreover, a ground electrode G is used to reduce the stimulus artifact. The electrodes for bipolar stimulation are also shown, in proximal and distal locations along the median nerve. The panel B of the figure shows a model of CB: in the case of healthy subjects all axons are intact, whereas in patients with multifocal demyelination (as in CIDP and MMN) there are some lesions that could block the propagation of the APs. Some examples of experimental thenar CMAPs are shown in C: a small delay between distal and proximal CMAPs is noticed in the healthy subject; a larger delay is noticed in the case of the CIDP patient with a reduced amplitude of the proximal CMAP; in the MMN patient, the proximal CMAP is widely spread and largely affected by phase cancellations, losing the biphasic shape.

**Figure 2:**
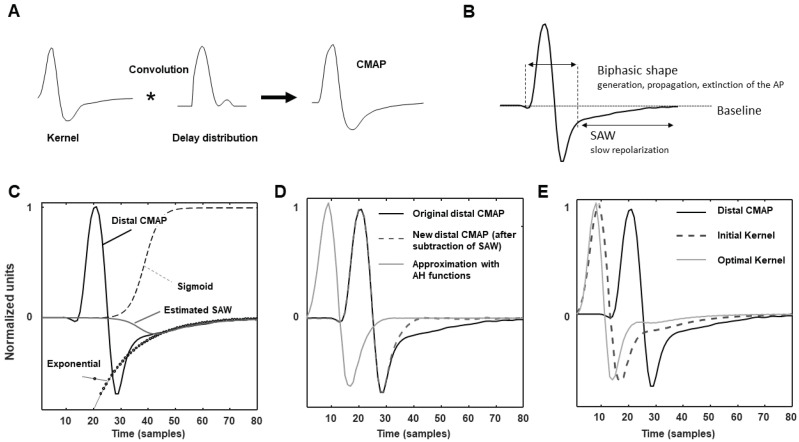
Representation of convolution (indicated by ∗) and example of kernel reconstruction, focusing on the slow afterwave (SAW). (**A**) A CMAP is represented as the convolution of a kernel and a delay distribution (both unknown). (**B**) Indication of the contribution of SAW in a CMAP. (**C**–**E**) Estimation of the kernel. (**C**) The SAW of the distal CMAP is estimated as the product of an exponential and a sigmoid function. (**D**) The estimated SAW is subtracted and the distal CMAP is reconstructed using AH functions (the reconstruction is time shifted for simpler representation). (**E**) The initial kernel is optimized to reduce the reconstruction error for the CMAP pair (including proximal and distal CMAP).

Figure 2 describes the deconvolution method, focusing on the estimation of the SAW, which is the main innovation introduced and the key to apply our approach to patients showing an important effect of the slow repolarization. The panel A is a sketchy representation of convolution, which recovers the CMAP by convolving a kernel and a delay distribution. The SAW is indicated in B in a CMAP: notice the slow return to baseline after the second phase. The panels (C–E) (bottom) show how SAW was estimated as the product of an exponential and a sigmoid function. As explained in the Methods section, the sigmoid was fit to the distal CMAP, which is less affected by TD (C). Then, the SAW was subtracted and the distal CMAP was reconstructed with AH functions (D). Finally, the initial kernel was iteratively updated by an optimization algorithm which uses it to fit both CMAPs (distal and proximal; the latter is not shown), obtaining at last the optimal kernel (E). Notice that it is shorter than the initial one, which was fit to the distal CMAP, which is a bit affected by TD.

**Figure 3:**
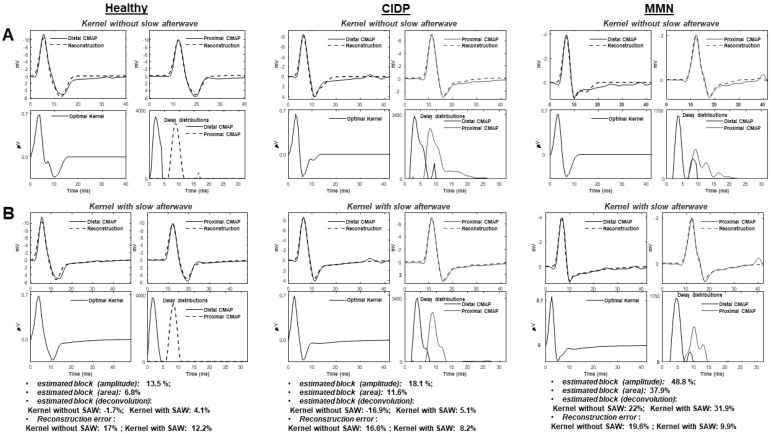
Comparison of deconvolution methods either excluding (**A**) or including (**B**) the SAW in estimating the kernel. Examples of data from healthy, CIDP and MMN subjects are considered. The estimation of CB is given in all cases, including also the approaches based on amplitude and area.

**Figure 4:**
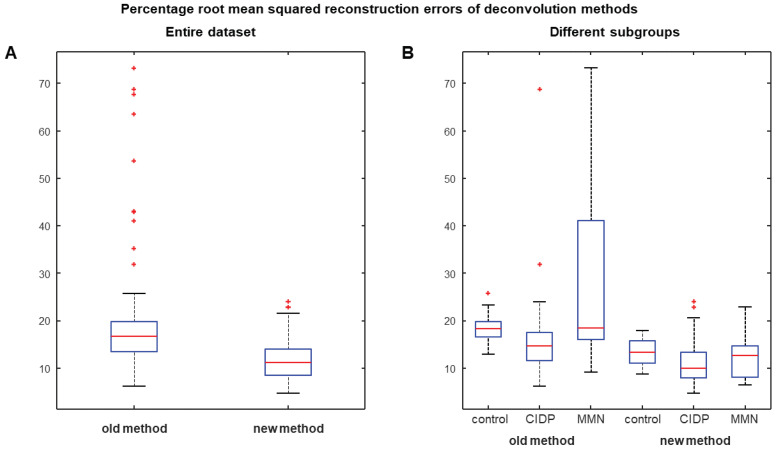
Comparison of the percentage reconstruction errors of the deconvolution methods either excluding or including the SAW in estimating the kernel (old and new method, respectively). Box and whiskers plots are displayed, showing median, quartiles and range (outliers indicated individually by red +) for (**A**) the entire dataset and (**B**) the groups of patients. All paired comparisons (either of the entire dataset or of each group of patients) are highly statistically significant under the Wilcoxon signed-rank test.

Figure 3 shows a comparison between deconvolution methods with kernel estimated either neglecting or including the SAW potential: (A) the previous algorithm proposed in [19]; (B) the new method. Examples of processing of different experimental data are provided. Better reconstructions of both healthy and pathological CMAPs were obtained by including a component modeling the slow repolarization of muscle fibers (reconstruction errors indicated below the figure). Considering the entire database (Figure 4), the new algorithm allowed to improve always the reconstruction (as it has additional degrees of freedom) with an average reduction of the root mean squared error of about 70%. Most problems are found by the old method when considering CMAPs of MMN patients, showing in the average larger TD values. Notice also that, considering a kernel neglecting the SAW (Figure 3A), the slow return of the CMAPs to baseline is poorly approximated by summing delayed contributions, which reflect into estimated delay distributions with a not reliable final portion. An inaccurate CB estimation is then expected. The problem is more evident in patients showing CMAPs with an important effect of the SAW.

**Figure 5:**
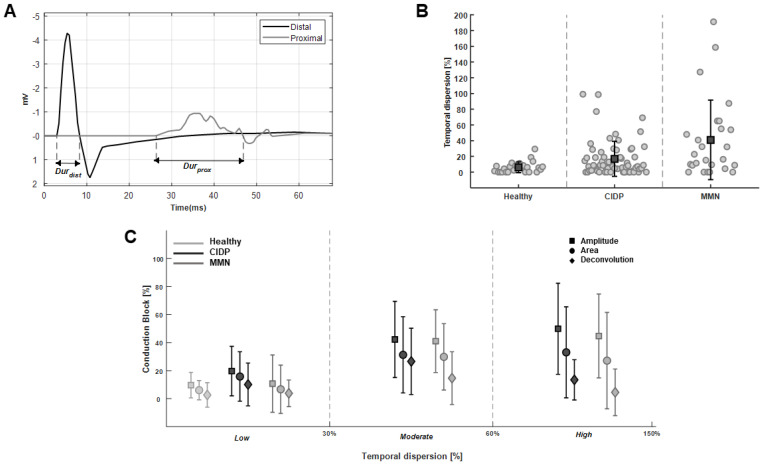
Investigation of temporal dispersion (TD) of CMAPs. (**A**) Definition of the measurement of the duration of the CMAPs in a case recorded from a MMN patient. (**B**) TDs of the CMAPs of our dataset (individual values indicated by circles; mean indicated by a square and standard deviation by the length of bars), grouped with respect to the pathology. (**C**) CB estimation with either standard (amplitude and area) or deconvolution methods, as a function of TD degree.

Figure 5 shows the effect of the TD on CB estimation. An abnormal TD in the CMAPs recorded from a MMN patient is shown in Figure 5A. The CMAP duration was computed as in the literature [44], considering the time interval between the onset and the last zero crossing from positive to negative phase. The TDs of the CMAPs for different pathologies are shown in Figure 5B. Not all nerves of patients had the same severity of the pathological manifestation, so that TD could widely vary. However, MMN patients, as a result of the specific neuropathy features, show a highly statistically larger TD than controls (*p* = 0.0012) and a statistically larger TD than CIDP signals (*p* = 0.025; Wilcoxon rank sum test); moreover, CIDP patients have a larger TD than controls (close to the limit of significance: *p* = 0.051). Differences are obviously emphasized by removing data from nerves of patients showing no effect of the pathology, e.g., with TD values lower than 10% (which happens in the 38% and 57% of our data from MMN and CIDP patients, respectively). In such a case, controls have a lower TD than patients (with *p*-values in the order of 10${}^{-7}$) and MMN patients still have larger TD than CIDP (with *p* = 0.041). CB estimation is shown in Figure 5C, comparing standard approaches (i.e., based on amplitude or area) and deconvolution, as a function of the degree of TD. The difference between standard and deconvolution methods increased proportionally with TD, as only the latter can discriminate between CB and TD. Indeed, the correlation coefficients of TD and CBs were 39.3%, 29.5% and 8.2%, for the amplitude, area and deconvolution methods, respectively (considering the old deconvolution approach neglecting SAW, the correlation between TD and CB was 27.4%, as it can compensate TD only if it is small, as in the case of healthy controls).

**Figure 6:**
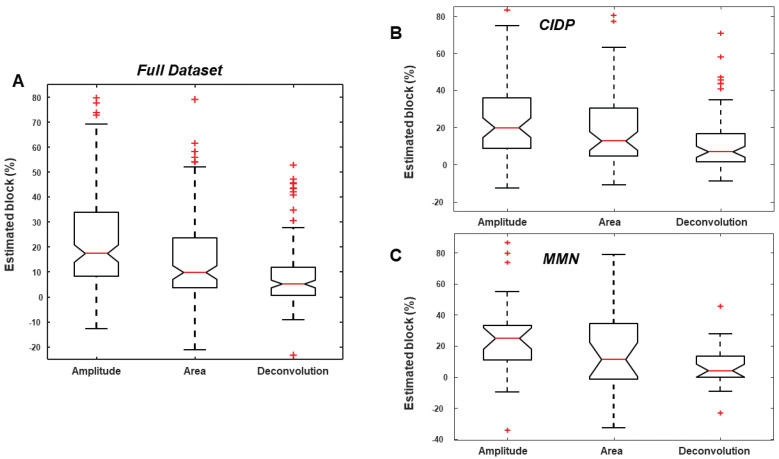
Analysis of variance of CB estimates obtained with standard and deconvolution methods on our dataset. Two way ANOVA on the full dataset indicated a significant difference between methods and pathology. (**A**) CB estimated by amplitude, area and deconvolution on the entire dataset (shown as box and whiskers plots, indicating outliers as red +). Panel (**B**) same as panel (**A**), but focusing on CIDP patients. (**C**) CB estimations on MMN patients.

Figure 6 shows the distributions of CB estimations on our entire dataset obtained with standard methods and with the new deconvolution approach. Scheirer–Ray–Hare two-way ANOVA of the full dataset indicated a highly significant effect of the methods and of the pathology (*p* < 0.001). Wilcoxon signed rank tests for the comparison of CBs estimated by different methods indicated highly statistical differences between all possible pairs (with negligible *p*-values). Wilcoxon rank sum tests indicated the following statistically significant differences between groups (*p* < 0.05): control and CIDP estimated by amplitude and area methods; control and MMN estimated by amplitude approach. No statistical difference in CB estimations was observed between the considered patients, even if MMN patients are better separable from CIDP when using the CB estimated by the proposed deconvolution method (Fisher’s ratio equal to 0.066, 0.002 and 0.003 for deconvolution, amplitude and area methods, respectively). The CB estimations are less dispersed when using deconvolution and they are not statistically different among groups. Notice that, TD was statistically different in different groups, so that CB estimated by amplitude and area approaches, being biased by TD, are facilitated in obtaining significant differences among groups. Moreover, as mentioned above, there were CMAPs recorded from some nerves of patients which did not show any pathological anomaly. Removing the data from pathological patients with TD lower than 10%, the above-mentioned results were emphasized. Moreover, the following additional differences were significant: control and CIDP estimated by deconvolution (*p* = 0.03); control and MMN estimated by area approach (*p* = 0.03). Differences between CIDP and MMN patients were not significant, but slightly better discrimination was obtained using the deconvolution method: Fisher’s ratio was equal to 0.078, 0.0004 and 0.0003 for deconvolution, amplitude and area methods, respectively.

## 4. Discussion

Nerve conduction studies are important to diagnose neuropathies. However, the standard clinical methods could be improved [45], reducing the confounding factors, mainly related to the interplay between CB and TD.

A new method is introduced to estimate CB and to distinguish it from TD in neuropathic patients. It is a generalization of a previous deconvolution method [19] that was shown to overcome the accuracy of the estimation of simulated CBs obtained by amplitude and area methods [19]. Moreover, deconvolution provided a CB estimation that was more stable to a variation of TD induced in experiments by increasing the conduction distance on the same healthy subjects [19]. The new method includes a model of SAW potential in the kernel (Figure 2), which is essential to allow for the reliable application of our deconvolution approach to patients. In fact, only generation, propagation and extinction of APs have been considered in modeling MUAPs in [19], neglecting the slow repolarization [20,21,22,23,24,25]. This part could be neglected when TD is small (as in healthy controls, studied in [19]), but it has a great importance in CMAPs from patients affected by demyelination. This has hampered the application of our previous deconvolution technique on patients and has stimulated the introduction of the innovative method discussed here, which has incorporated an exponential decay (fitting SAW) as additional term in the convolution kernel. The new algorithm outperforms the previous approach in approximating the proximal and distal CMAPs (with an average decrease of the reconstruction error of ~70%, Figure 4) and allows to estimate more reliable delay distributions and stable CBs (Figure 3).

### 4.1. Significance

Deconvolution allows to compensate for phase cancellations that largely affect proximal CMAPs of patients with large TD (due to the slowing of the APs of demyelinated axons; Figure 5). The proposed method provides a precise estimation of CB which is not biased by TD (Figure 6). Discriminating among TD and CB is fundamental to refine the clinical picture and to design properly the treatment. Moreover, an estimation insensitive to TD could allow to redefine the diagnostic standards for the assessment of CB [27,30], which are still largely conservative in order to face the possible bias affecting amplitude and area approaches [9,18]. A more accurate and robust estimation of CB could also allow for tracking the patient’s response to a treatment or for better stratification of different patients.

### 4.2. Limitations

Our results should be carefully interpreted keeping in mind some concerns due to model approximations, small dataset and limited information on the patients.

Specifically, notice that the number of elicited MUs can only be approximately estimated (and therefore also CB), as the recorded CMAP is affected by MUAP amplitude, which depends on MU dimension and location with respect to the recording system (in particular, there is a dependence on MU depth). However, also CMAP amplitude and area are affected by the same problem. Thus, even without solving all possible problems, deconvolution still has the great advantage of being (ideally) insensitive to TD over approaches based on CMAP amplitude and area. Moreover, the estimation of the delay distribution has the potential of investigating the type of MUs lost (e.g., predominately fast or slow or spread among different MUs).

Concerning the dataset, we should acknowledge that some problems arise from the retrospective nature of the study. For example, only a few data were available in electronic format, whereas many CMAPs have been manually converted into the digital form needed for processing, inevitably introducing some noise. As the investigated pathologies are rare, all available data have been included (still gathering a small and unbalanced dataset).

Moreover, many clinical records were not complete: thus, some precious information about conduction distance and location of stimulation/recording electrodes was not always available (so that reasonable estimations of conduction distance and electrode locations were assumed, as they were needed to impose physiological constraints on delay distributions; furthermore, on a few cases, those estimations were finely tuned to get good reconstruction of the CMAPs). Moreover, the actual condition of the different segments was not always available: it is indeed reasonable to suspect that nerves with normal axonal conduction did not show any sign of the pathology, whereas the corresponding CMAPs where included in the group of the patient’s diagnosed pathology (hindering discrimination of different groups).

### 4.3. Future Perspectives

Being stable to TD, our method could diagnose CB with a lower confidence threshold than for amplitude and area approaches. Moreover, a precise measure of CB could allow the follow-up of a patient and the study of the response to a treatment. Possibly, the estimation of CB could also be correlated to the actual symptoms in a prospective study.

In addition, more information than merely CB could be extracted from the delay distributions. For example, the spread of CVs of the axons of motor neurons could be investigated and some information on the types of MUs affected by a possible block could be retrieved. Moreover, a new estimation of TD could be proposed: indeed, the present definition is based on the shape of the CMAPs, which is affected by phase cancellations, whereas the delay distributions are not. Investigating if the estimated delay distributions, even being approximated (as they are based on modeling assumptions and affected by experimental noise), could provide reliable information is a future work with promising prospects.

## 5. Conclusions and Further Work

A new method for CB estimation has been developed and tested in a preliminary pilot cohort of patients. It compensates the effect of TD (which afflicts clinical approaches based on CMAP amplitude and area) and outperforms a state-of-the-art deconvolution method in approximating the CMAPs, thus providing more reliable information on delay distributions (from which CB is estimated). Further tests on a prospective clinical trial are needed in order to better assess its reliability and possible applications.

## Data Availability

Data available only on request, due to privacy restrictions.

## Abbreviations

The following abbreviations are used in this manuscript:

AP	Action Potential
CB	Conduction Block
CIDP	Chronic Inflammatory Demyelinating Polyneuropathy
CMAP	Compound Muscle Action Potential
EDX	Electrodiagnostic
EMG	Electromyogram
MMN	Multifocal Motor Neuropathy
MU	Motor Unit
MUAP	Motor Unit Action Potential
NEE	Needle Electrode Examination
NCS	Nerve Conduction Study
PNS	Peripheral Nervous System
SAW	Slow AfterWave
TD	Temporal Dispersion
